# Combining laser ablation and sol–gel techniques for the synthesis of nanostructured organic–inorganic matrices

**DOI:** 10.3762/bjnano.17.101

**Published:** 2026-10-23

**Authors:** Emmanuel Haro-Poniatowski, Cesar Augusto Guarín, Luis Guillermo Mendoza-Luna, Luis Escobar-Alarcón, José Luis Hernández-Pozos, L Irais Vera-Robles, Patricia Castillo, Fátima Cabello, Johann Toudert, Fernando Chacón-Sánchez, Marina García-Pardo, Rosalia Serna, José Gonzalo, Javier Solís

**Affiliations:** 1 Departamento de Física, Universidad Autónoma Metropolitana Iztapalapa, Apdo. Postal 55-534, México City, México; 2 Laser Processing Group, Instituto de Óptica, IO, CSIC, Serrano, 121 28006 Madrid, Spain; 3 Departamento de Física, Instituto Nacional de Investigaciones Nucleares, Carretera México-Toluca S/N, la Marquesa, Ocoyoacac, Estado de México. C.P.52750, México; 4 Departamento de Química, Universidad Autónoma Metropolitana Iztapalapa, Apdo. Postal 55-534, México City, México; 5 Laboratorio Microscopía Electrónica, Universidad Autónoma Metropolitana Iztapalapa, Apdo. Postal 55-534, México City, México

**Keywords:** laser ablation, nanocomposite glasses, sol–gel

## Abstract

In this work we report a new and simple method that combines pulsed laser ablation in liquids (PLAL) and sol–gel techniques to obtain nanocomposite glasses and gelatins. Using this combination, we obtained two kinds of materials, bulk glasses on the one hand and gelatin films on the other hand. In the case of glasses, gold nanoparticles (Au-NPs) are generated by PLAL using an Au target submerged in a transparent liquid solution made previously with tetraethyl orthosilicate (TEOS) adding diluted hydrochloric acid as catalyzer. For gelatins, the same procedure is employed; however, in this case only commercial gelatin and tap water are used. We have found that a successful synthesis strongly depends on time and temperature since the viscosity of the gels increases with time. For the drying process the gels are sealed and stored at room temperature for several days. We identify the principal parameters that have to be considered in order to achieve a successful synthesis. The samples are characterized by UV–vis spectroscopy, HRTEM, ellipsometry and AFM microscopy. UV–vis spectra show a surface plasmon resonance band with its maximum at 540 nm indicating the presence of Au-NPs. HRTEM reveals crystalline Au-NPs with an average size of 20 nm. AFM measurements indicate that the glasses have a very smooth surface. In the case of gelatins, self-sustained, transparent, and pink flexible films are obtained. Despite having determined the principal parameters involved in the obtention of these materials, we emphasize that a lot of work remains to be done in order to optimize this novel method of synthesis.

## Introduction

Nanocomposite glasses have been around us since ancient times. Nowadays we know that the change in color upon transmission and reflection of light of the exceptional fourth-century Lycurgus cup, fabricated by the Romans, is due to the fact that it is made of a glass with Au and Ag nanoparticles (NPs) embedded in it [1], forming a nanocomposite. A nanocomposite is a kind of material in which one phase with nanoscale dimensions is embedded in a ceramic, glass, or polymer matrix. Great effort has been made recently to incorporate NPs into a wide variety of matrices that can be liquid or solids, crystalline or amorphous. This incorporation results in changes in the physical and chemical properties of the matrices, which are in some cases better than the properties of their individual components. Some examples are the improvement of the tribological properties of metals, oils, and polymers [2–4]. Nanoparticles of CdTe of different sizes have been dispersed in liquid, changing their color progressively from yellow to blue according to their corresponding size [5]. Other applications include quantum computing, with significant efforts pursued in fabricating nanodots embedded in different matrices and structures [6]. Additionally, multiple applications in the field of plasmonics require the synthesis of nanostructures in various substrates or matrices [7]. Very recently, the idea of embedding nanoparticles into a glassy matrix has been extended to include gelatin as a matrix to fabricate eco-friendly optical components such as flexible filters [8].

For a few years, we have been involved in the synthesis and development of nanostructured glasses. Our interest has been focused on nanoparticles of metals with low melting point immersed in solid matrices with high melting point. These systems are important because of their potential applications in optothermal devices such as thermo-optical switches responding to changes in temperature [9–10]. However, only a few examples can be found in the literature regarding these nanocomposites. To our knowledge, only Se, Bi, and Pb nanocrystals, and more recently CuCl and CuBr have been synthesized successfully in bulk amorphous matrices [11–12].

Finally, it is important to mention that the process of making such glasses is difficult and time- and energy-consuming due to the high temperatures needed in their fabrication. Consequently, synthesizing such systems “on demand”, for example, the fabrication of a glass with embedded elemental metallic NPs, is not an easy and straightforward process. Several routes have been, and are currently, under investigation. Pulsed laser deposition (PLD) has proved to be a suitable method to fabricate such nanocomposites [13–15].

An alternative possibility for the fabrication of the desired colloidal systems is the pulsed laser ablation in liquids (PLAL) technique [16]. Nanostructures of different sizes and morphologies including metals, alloys, and oxides have been produced by this technique [17–18]. An important advantage of PLAL is its versatility, enabling its combination with other fabrication processes in an easy way. In such direction, we have synthesized carbon nanosheets using PLAL under an ultrasound field [19]. However, the reports of PLAL-made nanocomposites are scarce. As mentioned before, nanoparticles have been dispersed by PLAL in polymers and in resins. For example, nail enamels of different colors have been produced by dispersing Au, Ag, and Pt nanoparticles by PLAL in a nail varnish [20].

For the synthesis of nanocomposite glasses, an alternative and convenient solution is the sol–gel technique. This method has the advantage of being fast and simple; furthermore, the materials are fabricated at room temperature, and eventual thermal treatments are of the order of only a few hundred degrees Celsius. The main drawback is that it is not easy to obtain large monoliths. Furthermore, the drying process is long and difficult to optimize. Using this technique, nanostructured glasses have been obtained by mixing organo-metallic products and colloids with nanoparticles. However, the variety of organo-metallic products has some limitations such as availability and price. Furthermore, an important advantage of PLAL is its independence from chemical precursors, avoiding the use of toxic substances or other by-products that can be present in the obtained material. To the best of our knowledge, there are few works that combine PLAL with a chemical route for synthesis of nanocomposites. The coating of gold nanoparticles with silica shells via PLAL in a single-step by ablating a gold target directly inside a mixture of tetraethyl orthosilicate (TEOS) and a catalyst has been reported [21]. Gd_2_O_3_@SiO_2_ nanoparticles, formed by a gadolinium oxide core and a silica shell, have been synthesized in a single step via PLAL of a submerged Gd_2_O_3_ target in TEOS. As Gd_2_O_3_ nanoparticles are formed, they are immediately coated with SiO_2_, directly yielding a core–shell structure for the use as MRI contrast agents [22].

In the present work, we propose to use the combination of the sol–gel and laser ablation techniques to synthesize nanostructured glasses and gelatins [23]. The main requirement is to use a solution that is transparent at the wavelength used for ablation. The variety of glassy materials and gelatins that can be fabricated is very broad, while possible limitations could be the availability of the solid target, or the nature of the solvent. In the first approach of this combination of techniques, we have obtained Au nanoparticles embedded in a silica matrix and in a commercial gelatin. The presence of the NPs has been confirmed by UV–vis spectroscopy and TEM microscopy. The synthesis of the nanocomposite glasses reported in the present work is fast and simple compared to other methods of fabrication and has several potential applications since the materials can be prepared in bulk and thin film forms. However, a lot of work is still needed to optimize several parameters that determine the obtention of the final product. In the case of gelatins, we present some preliminary results in Supporting Information File 1, Section S1. The fabrication process is simpler than that of sol–gel glasses, and the results are very similar to those reported in [8] except for the method of the nanoparticles incorporation in the gelatin, which is PLAL in our case.

## Results and Discussion

First, the optimum experimental conditions must be determined. The main parameters involved in the synthesis of nanocomposite glasses combining PLAL and sol–gel techniques are the following: (a) transparency of the sol, (b) temperature, (c) viscosity (viscosity decreases with temperature), (d) ablation time (viscosity increases with time), (e) amount of liquid above the target, (f) energy density, (g) laser wavelength, and (h) post-preparation thermal treatments. Furthermore, the optimization of these parameters in some cases can involve the change of the original formula used for the preparation of the sol as discussed later. Most of these parameters have been identified in previous works [20,23]. As said before, a very sensible one is viscosity, since it is directly related to the proper dispersion of nanoparticles; this aspect needs to be thoroughly investigated in future experiments.

### Sol–gel glasses

**Figure 1:**
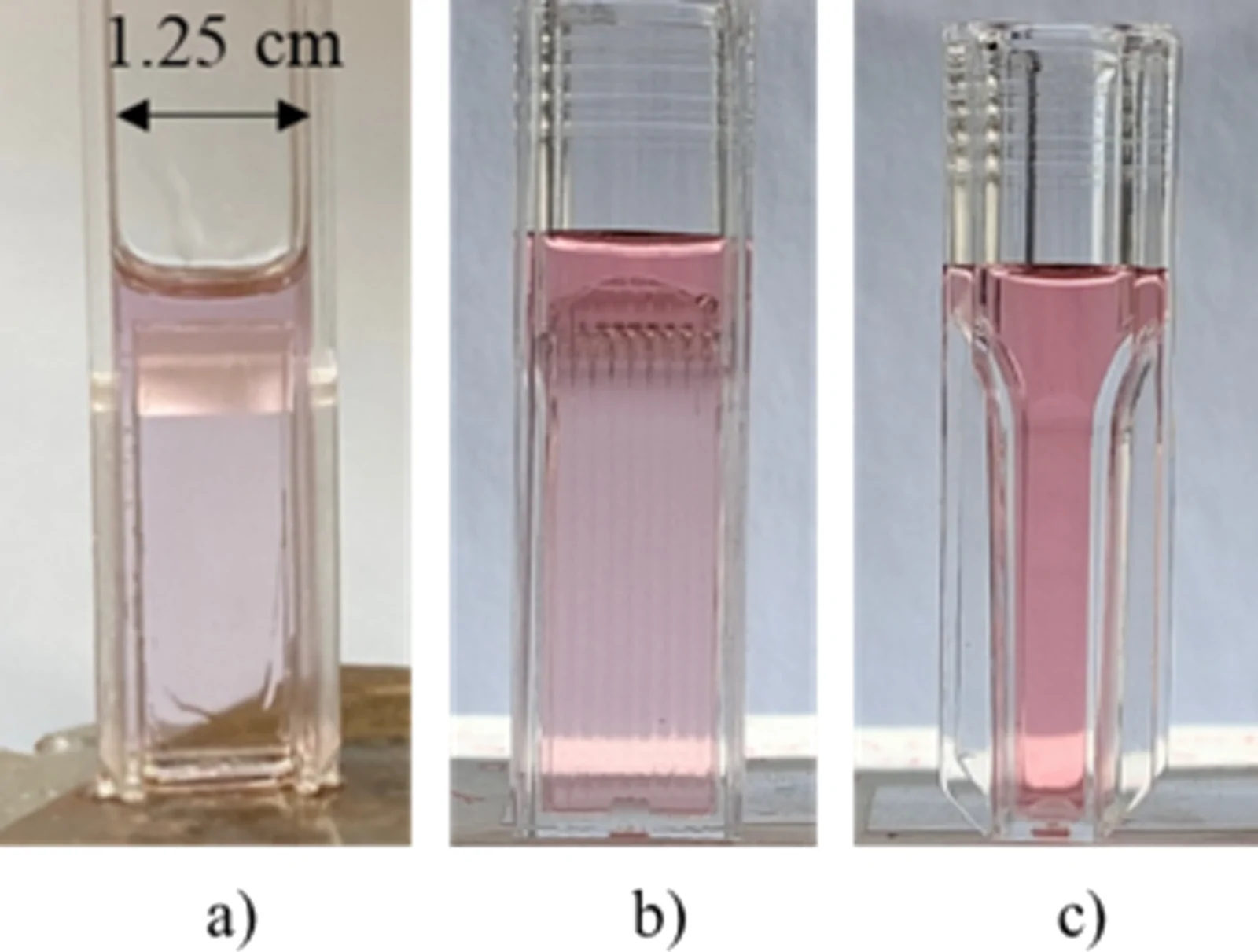
(a) As-prepared Au-NPs in the sol–gel solution, (b) Au-NPs in distilled water, and (c) lateral view of (b).

The ablation process starts typically within one hour after the preparation of the sol when its temperature is around 40 °C; this gives enough time to perform the ablation (10 min) and to obtain a high dispersion of the Au nanoparticles. The effect of increasing viscosity with time is easily observable by the eye; however, a suitable experimental procedure is needed for obtaining quantitative information. This investigation is out of scope of the present work. The amount of liquid is also crucial for the laser ablation process, a thin layer of liquid results in significant splashing. In the present experiments, we have used a liquid layer around 1 cm above the surface of the target. As soon as the ablation process takes place, the production of gold nanoparticles can be detected visually. In Figure 1, for coloration comparison, the obtained gel is presented (Figure 1a) together with a colloid of gold nanoparticles (Au-NPs) obtained by PLAL in distilled water (Figure 1b) under the same experimental conditions.

**Figure 2:**
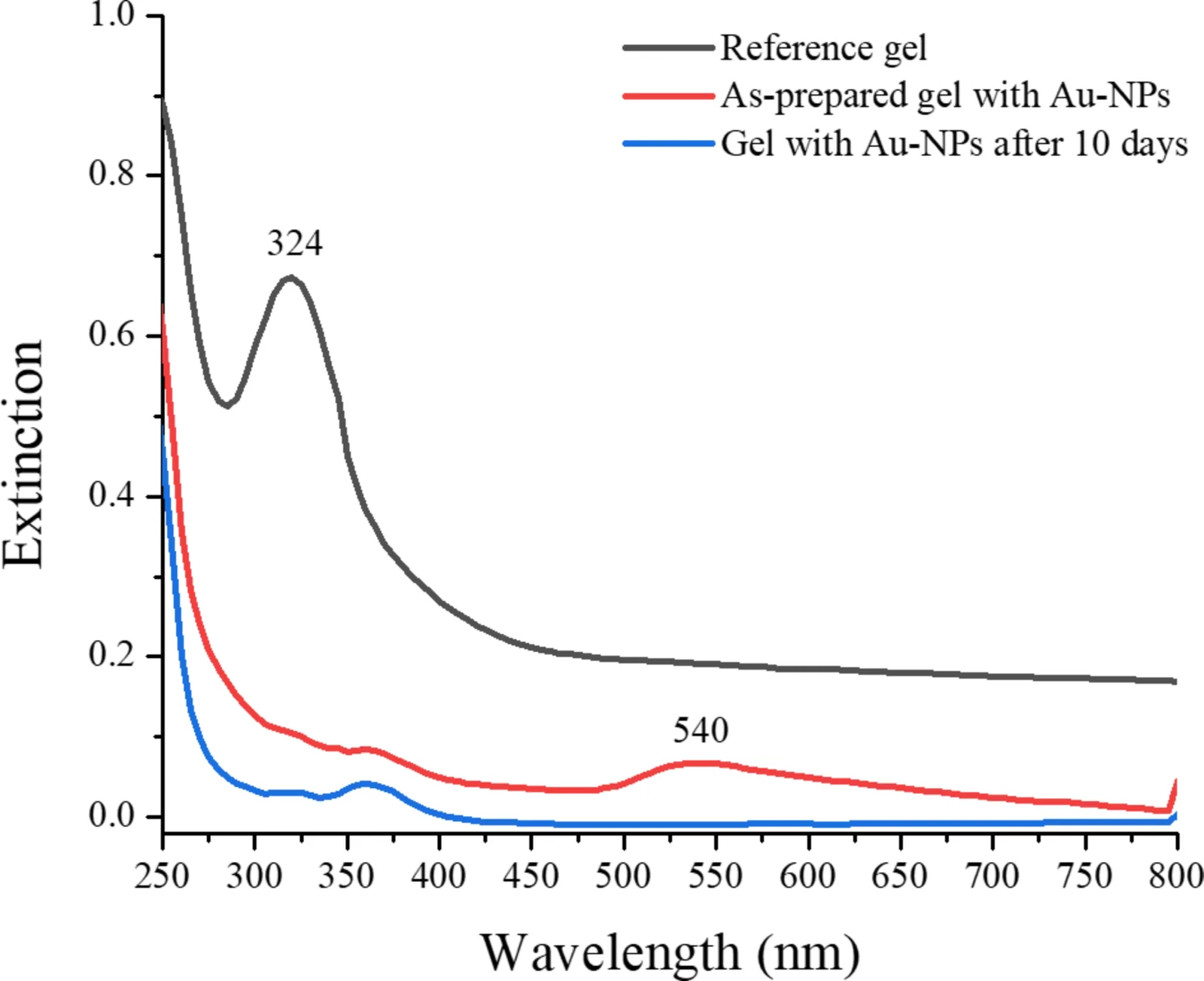
Extinction spectra of the reference gel without Au (black curve), the sol–gel obtained after the ablation of the Au target (red curve), and the same sample with Au-NPs after 10 days (blue curve).

The gels were immediately characterized by UV–vis spectroscopy. The same setup configuration and measurement method were used for all the UV–vis spectroscopy measurements reported in this paper. Before ablation, a transparent reference sample was withdrawn from the sol. The UV–vis extinction spectra of the reference gel, the as-prepared gel with Au-NPs, and the gel with Au-NPs after ten days of drying are presented in Figure 2. The blue spectrum corresponds to the reference sample; as expected, no extinction features are observed in the visible range. The spectrum depicted in red shows a band around 540 nm that reveals the presence of Au-NPs in the gel and is attributed to the surface plasmon resonance of Au. The 540 nm resonance wavelength corresponds to NPs with a non-retarded dipolar resonance. If we assume, as a first approximation, that the NPs are monodisperse with spherical shape and that the refractive index of the surrounding medium is 1.44, Mie calculations (Supporting Information File 1, Section S2) suggest that the 540 nm peak position corresponds to NP diameters between 40 and 50 nm. Note also that the measured plasmon band spans the range from 520 to 800 nm and displays an asymmetric shape with a broader extension toward longer wavelengths. This contrasts with Mie simulations for a given NPs size and suggests that the NPs have a broad polydisperse size and/or shape distribution, with bigger and/or non-spherical NPs resonating on the long wavelength side of the spectrum [24]. Finally, from effective medium simulations (Maxwell–Garnett model) of the measured extinction spectrum, we estimate the volume fraction of Au-NPs to be very small, in the 10^−4^% to 10^−3^% range.

The black spectrum in Figure 2 was recorded 10 days after preparation. The striking feature in this spectrum is that the band corresponding to the well-defined Au plasmon resonance has disappeared and an intense absorption band peaking at 324 nm appeared. Here we provide a preliminary discussion about why this plasmon band has disappeared. While no well-defined plasmon can be seen in the spectrum, there is a broad background, which might result from the presence of bigger NPs, with sizes of hundreds of nanometers, that do not show well-defined plasmon resonances as supported by the Mie simulations shown in Supporting Information File 1, Section S2. In other words, the NPs might have formed bigger structures within the matrix, either by coalescence or Ostwald ripening. Another, yet less likely, possibility is that a significant amount of Au has migrated toward the surface where it formed big NPs, as has been reported in other studies for Ag in glasses and thin films [25–26]. One might also think that the Au-NPs have chemically reacted with the matrix (e.g., Au-OC_2_H_5_ complexes, the acid inducing oxidation of Au to Au^+^) or lost their plasmonic character due to intense chemical interface damping [16,27–28]. However, such effects usually have a strong impact only in the case of few-nanometer NPs, much smaller than in the present case. Note also that the appearance of the band at 324 nm is somewhat simultaneous to the disappearance of the Au plasmon band. This appearance is likely due to changes in the matrix as Au-NPs cannot display plasmon resonances in the UV. Further characterization, beyond the scope of this paper, is needed to definitely address the origin of the observed trends.

### Drying and shrinking processes

**Figure 3:**
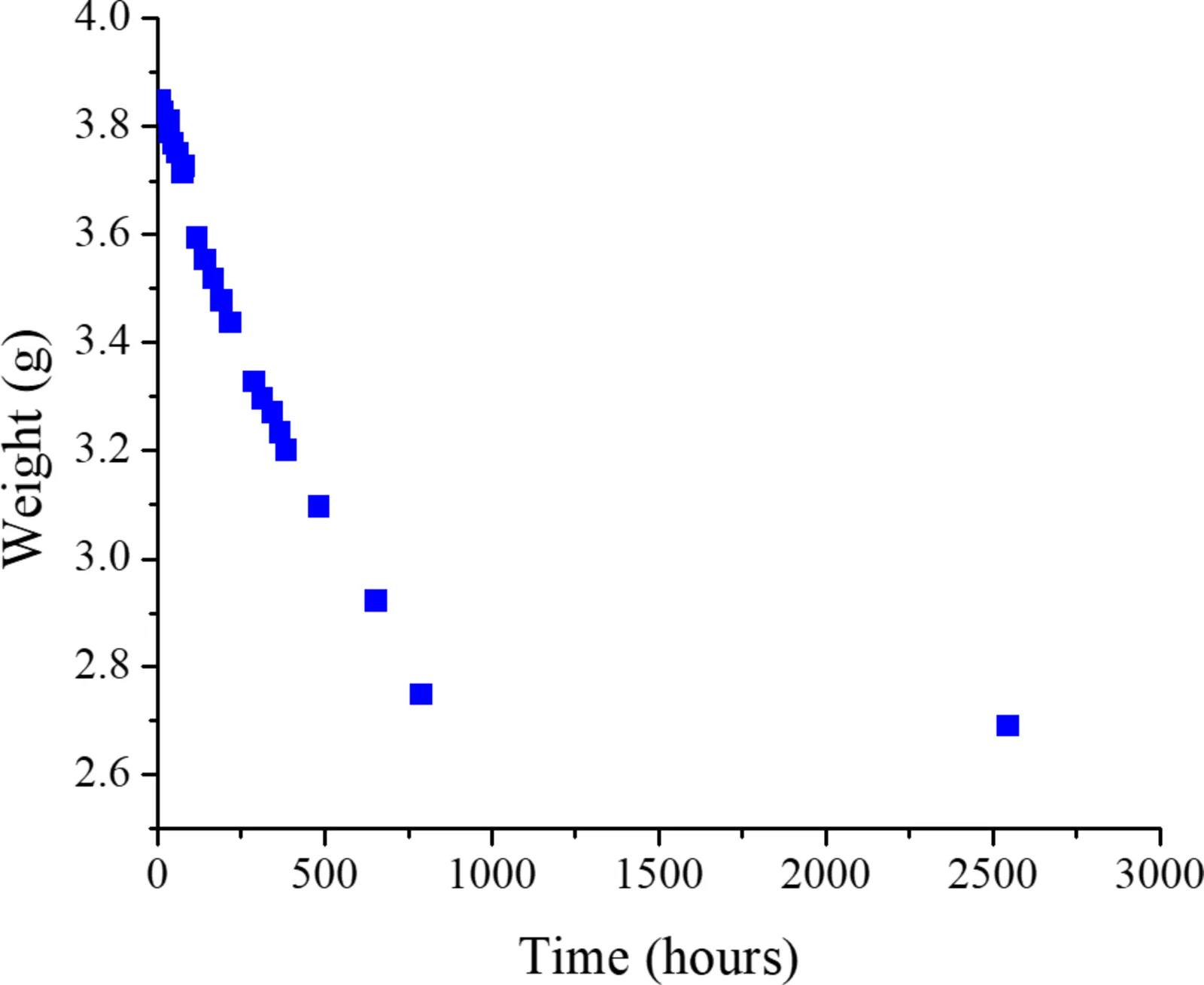
Weight of a sol–gel sample in a cuvette for UV–vis spectroscopy as a function of time.

The drying procedure appears to be crucial for the final result and is still under investigation. There are many publications regarding this aspect of the synthesis of sol–gel glasses [29–30]. Once the sol has been poured into the UV cuvette, the drying process starts. We have measured the weight loss as a function of time, as is shown in Figure 3.

**Figure 4:**
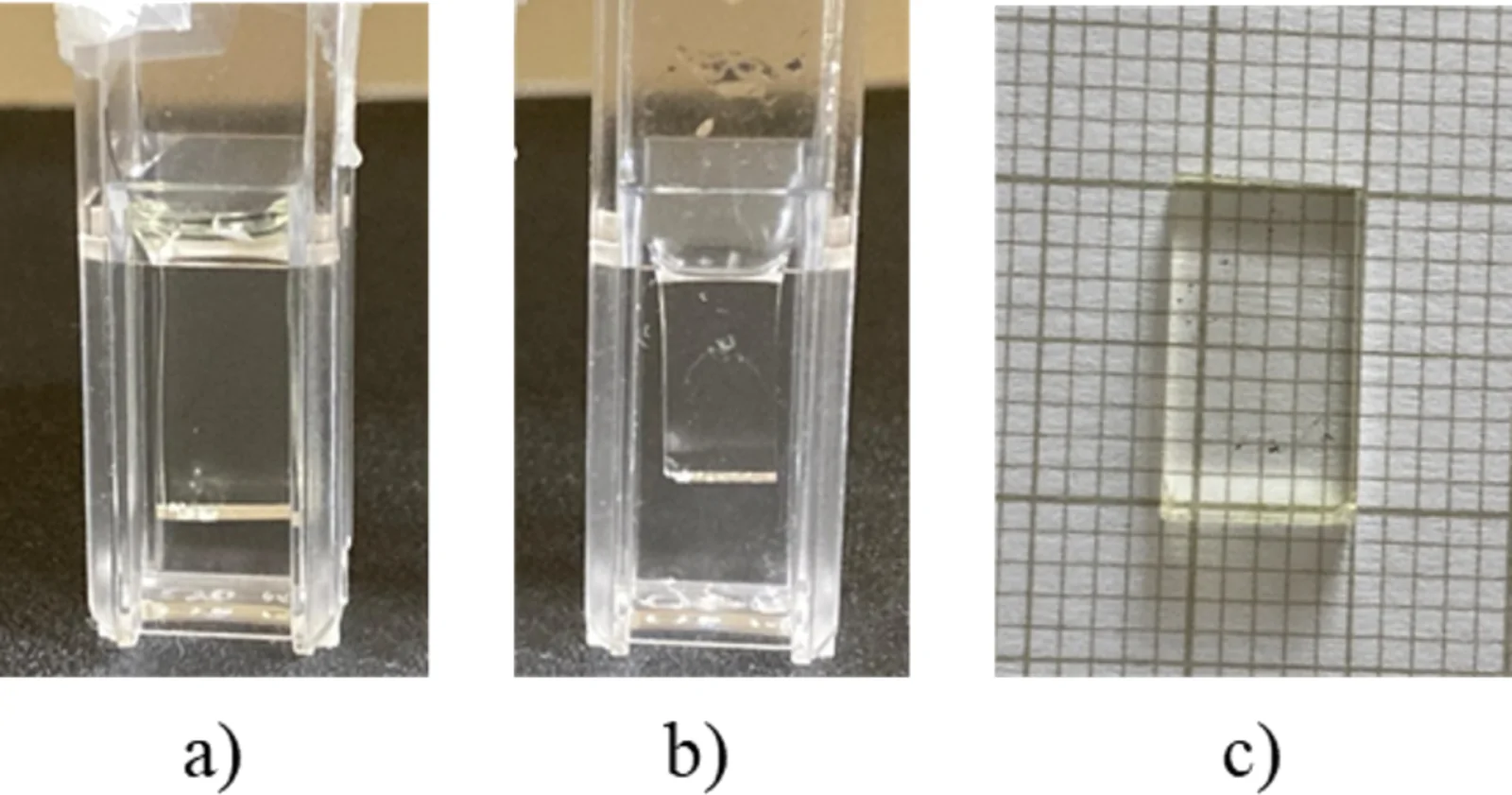
Shrinking process after (a) one month, (b) two months, and (c) three months of drying (images not in scale).

In the first hour, the evaporation occurs quite fast and if the vessel is not partially sealed, the probability of fracture once the material solidifies is very high. After approximately three months of drying, the sample has shrunk and lost about 30% of its original weight. We have tried to optimize the drying process by changing the vessels, which determine the morphology and size of the glasses, and the sealing procedure. The vessels are covered with a plastic film with some holes to allow evaporation. Best results have been obtained using UV–vis spectroscopy cuvettes designed for 1.5 mL sealed with parafilm with some small holes in it. Just after preparation, the sol–gel solution occupies most of the sample volume of the cuvette (photograph not shown here). In Figure 4 we show the drying–shrinking process. Figure 4a and Figure 4b show the sample one month and two months after preparation, respectively. The final product after three months and after some slight polishing of the top is shown in Figure 4c. It is a quite hard transparent block of glass, 6 × 2 × 10 mm^3^ in size. At this stage, the obtained sample is very robust and can be easily manipulated but it is certainly not completely dry. We have examined samples that have dried for up to two years; although the optical properties of the sample exhibit only small changes, we cannot guarantee that they are completely dry.

Regarding the final block shown in Figure 4c, an AFM image reveals a very flat surface as shown later. The only surface that must be polished is the upper one, which maintains the block vertically in the cuvette. In the case of gelatins, the drying and shrinking processes are described in Supporting Information File 1, Section S1.

### Thermal treatments

**Figure 5:**
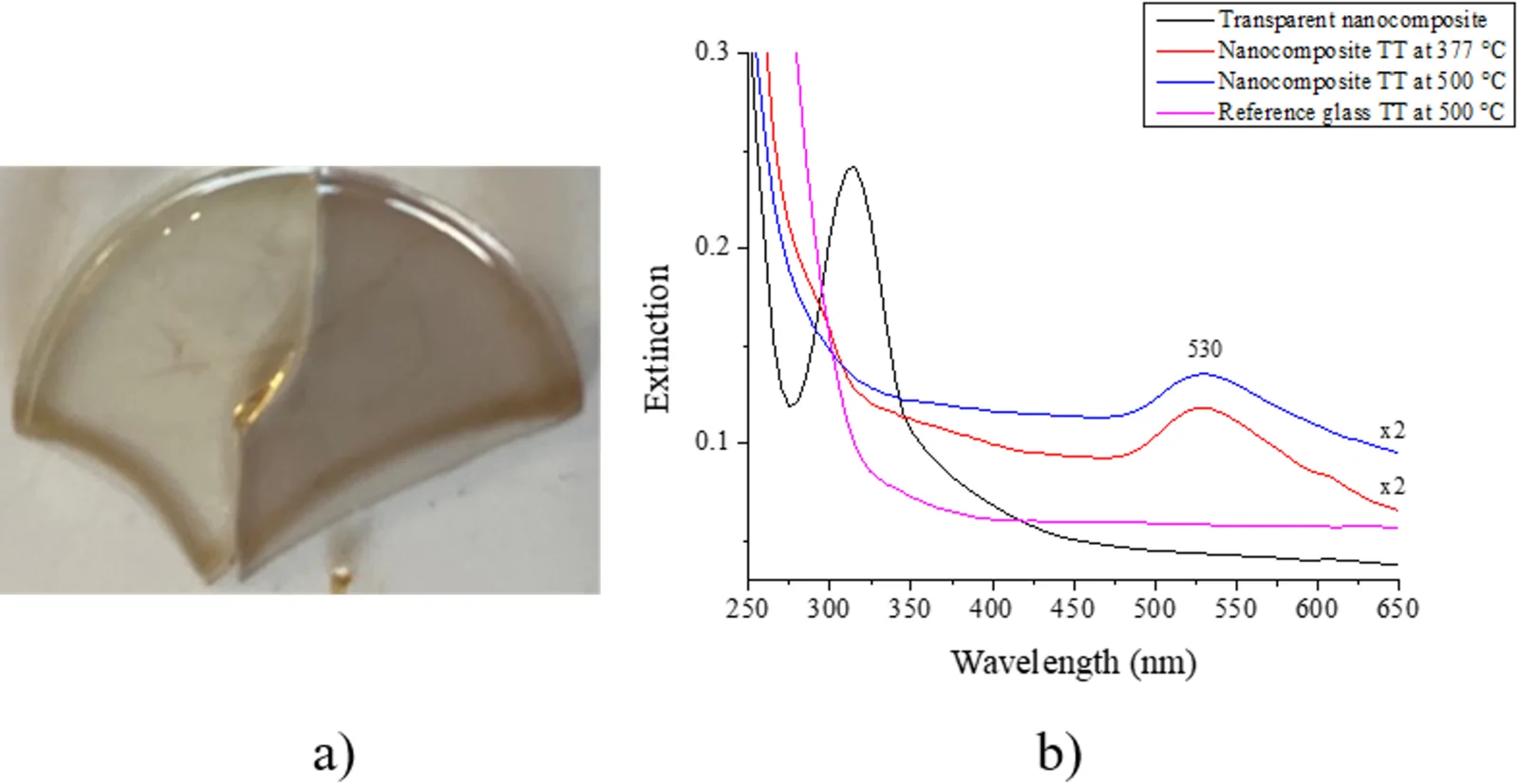
(a) Photograph of a piece of glass before (left) and after (right) thermal treatment at 377 °C, (b) extinction spectra corresponding to the transparent nanocomposite and after two thermal treatments (TTs) at 377 and 500 °C; a reference glass TT at 500 °C is included. Note that the spectra of the thermally treated samples (377 and 500 °C) are scaled (×2) to facilitate comparison.

In many cases, and depending on the preparation variables used for the synthesis of the nanocomposites, once they have almost completely dried in the form of blocks or thin flat pieces, they can be thermally treated to retrieve the original pink color and the characteristic Au plasmon resonance near 530 nm. Thermal treatments at heating rates ranging from 1 °C/min to 10 °C/min were used. Best results are obtained for slow heating rates and depend on the shape and thickness of the samples. The results and UV–vis spectra are shown in Figure 5. Figure 5a shows a photograph of a piece of glass before (left) and after (right) a thermal treatment at 377 °C for 5 h at a heating rate of 3 °C/min. Figure 5b shows the extinction spectra of a nanocomposite glass with Au-NPs before and after two thermal treatments (TTs), at 377 °C and 500 °C for 5 h each, at a heating rate of 3 °C/min. The Au plasmon at 530 nm is clearly visible over a spectrally broad background; the spectrum of a reference glass (without Au-NPs) after thermal treatment at 500 °C is included for comparison. This suggests that, while large, few-hundred nanometer Au-NPs dominate the broadband optical response of the annealed glass, the annealing has also led to the formation of small, few-nanometer Au-NPs, perhaps from nucleation growth involving atoms that remained isolated in the matrix prior to annealing. The reappearance of the plasmon band also indicates that the Au-NPs that have chemically reacted during ablation [16,28] and revert their oxidation state upon thermal annealing.

**Figure 6:**
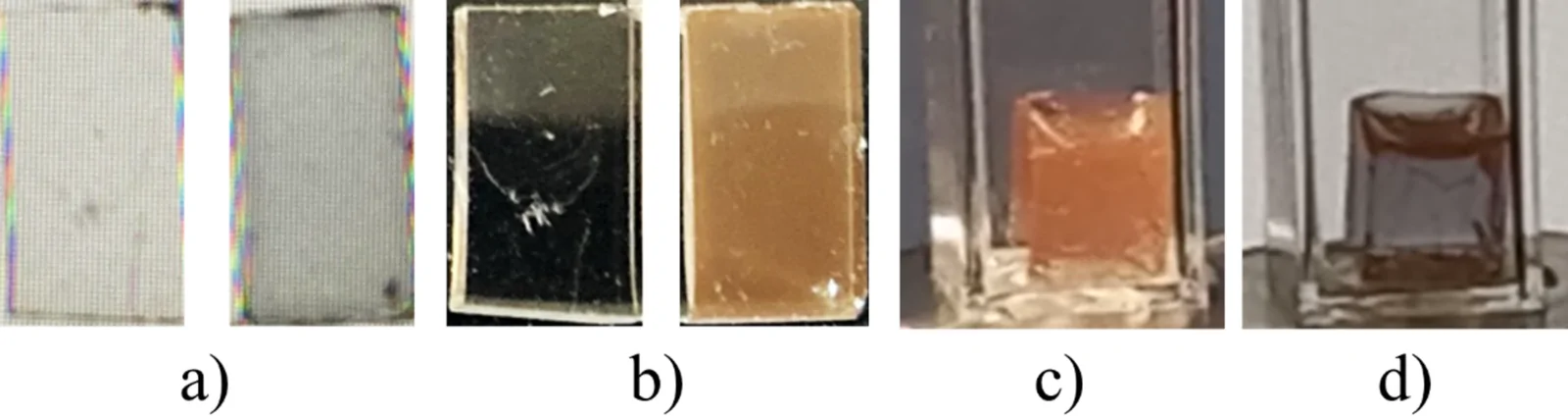
Photographs in transmission and reflection illumination of (a) a reference and (b) a sample with Au-NPs that have dried for six months. A nanocomposite with Au-NPs after two years of drying illuminated in transmission (c) and reflection (d).

Furthermore, we have observed that, after a very long drying process, the original plasmon resonance peak remains. In Figure 6 we show photographs of a reference sample (Figure 6a) and a nanocomposite with gold nanoparticles (Figure 6b) that has dried for six months. The sample on the left side is illuminated in reflection whereas the sample on the right side is illuminated in transmission. For the sample containing Au-NPs, the change in color is evident. Figure 6c and Figure 6d show a sample after two years of drying in transmission and reflection illumination respectively resembling the well-known dichroic effect observed in the Lycurgus cup. In Supporting Information File 2, we show a video to illustrate this color change in real time for the sample that has dried for two years. As mentioned before, a thermal treatment helps to accelerate the nanocomposite color recovery which is very slow if the glass is left to dry for a long time.

### HRTEM characterization

**Figure 7:**
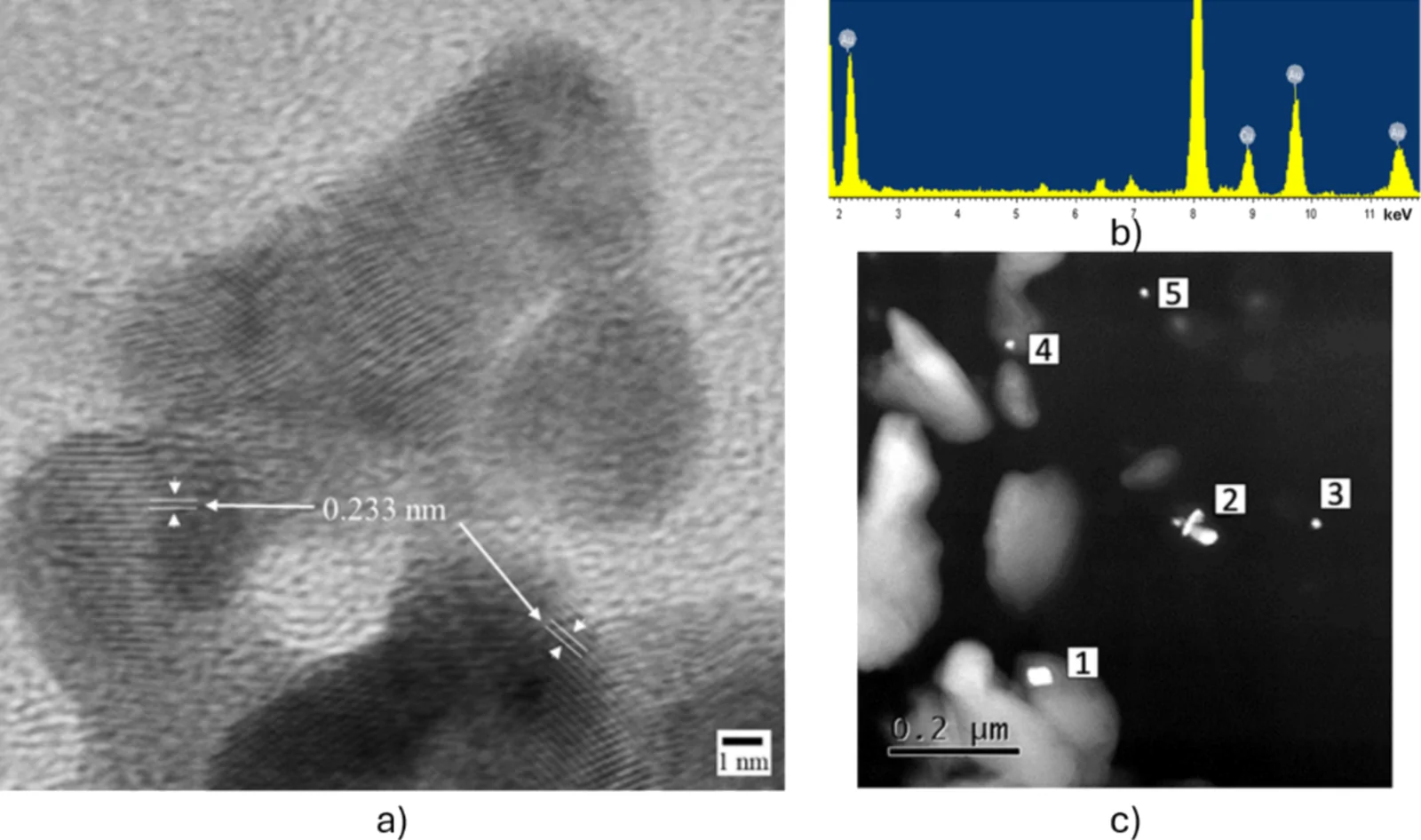
(a) HRTEM image of a nanocomposite sample after two years of drying, (b) EDS spectrum of one Au-NP, (c) HAADF-TEM image showing the presence of Au-NPs.

To confirm the presence of Au-NPs in the obtained nanocomposite glasses, we have obtained HRTEM images. Figure 7a shows a high-resolution image in which nanoparticles with an interplanar distance of 0.233 nm, corresponding to the (111) planes of gold, are observed. The EDS spectrum shown in Figure 7b confirms that these are Au nanoparticles. A sample was analyzed by high-angle annular dark-field (HAADF) technique and from some images (Figure 7c shows one of them), sizes ranging from 10 to 43 nm in diameter were measured with an average of 20 nm, determined by analyzing 22 Au-NPs.

### AFM characterization

**Figure 8:**
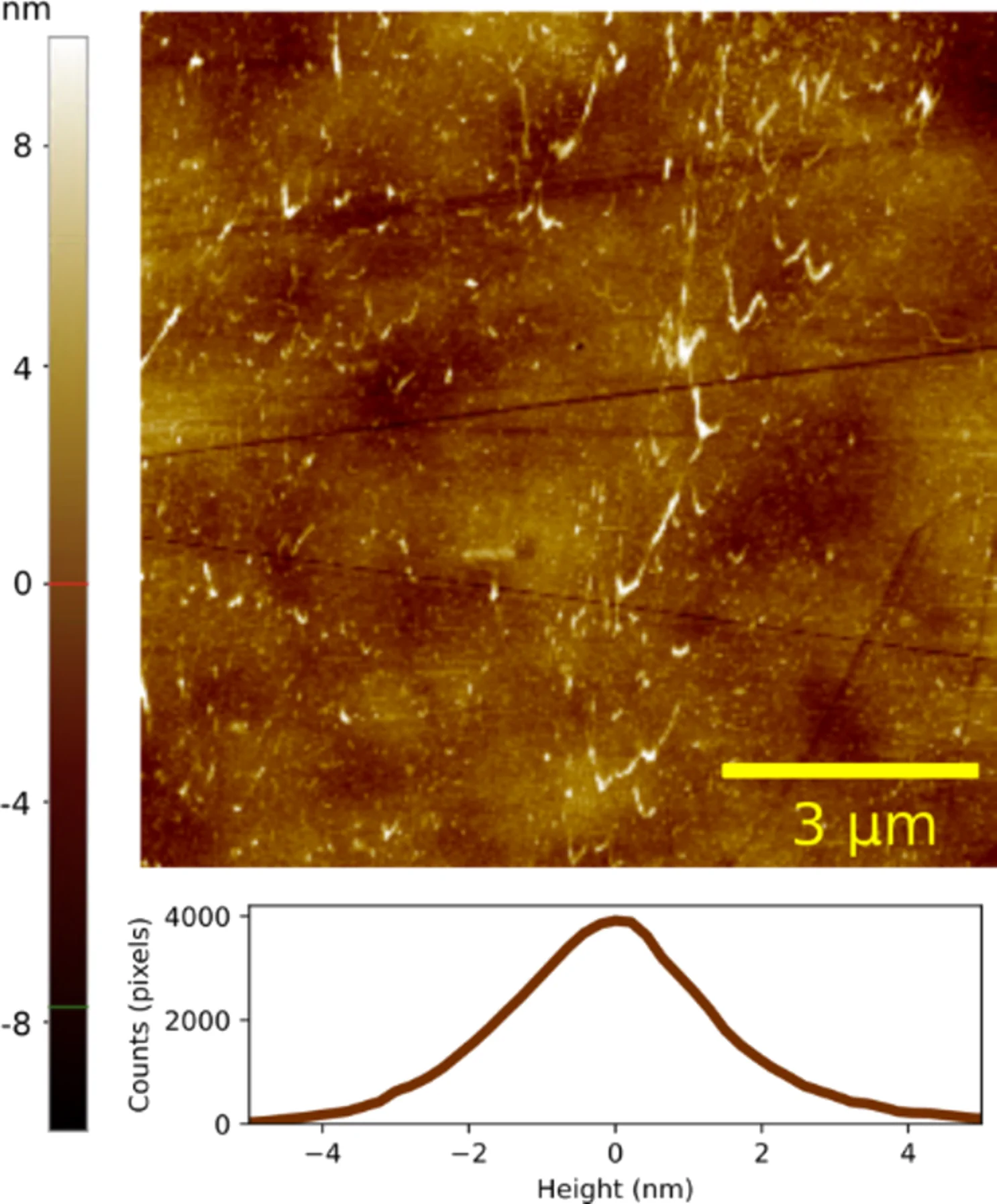
AFM image of a pure glass sample after six months of drying.

To characterize the surface topography of the synthesized glasses, AFM measurements were performed via non-contact mode measurements. In Figure 8, we can see the smoothness of the surface, with most height values in the ±4 nm range and a mean square roughness of 1.92 nm. This confirms that the glasses have a smooth surface that will not play any role in the optical characterization, as well as opening a pathway for applications as a functional substrate.

### Optical characterization

**Figure 9:**
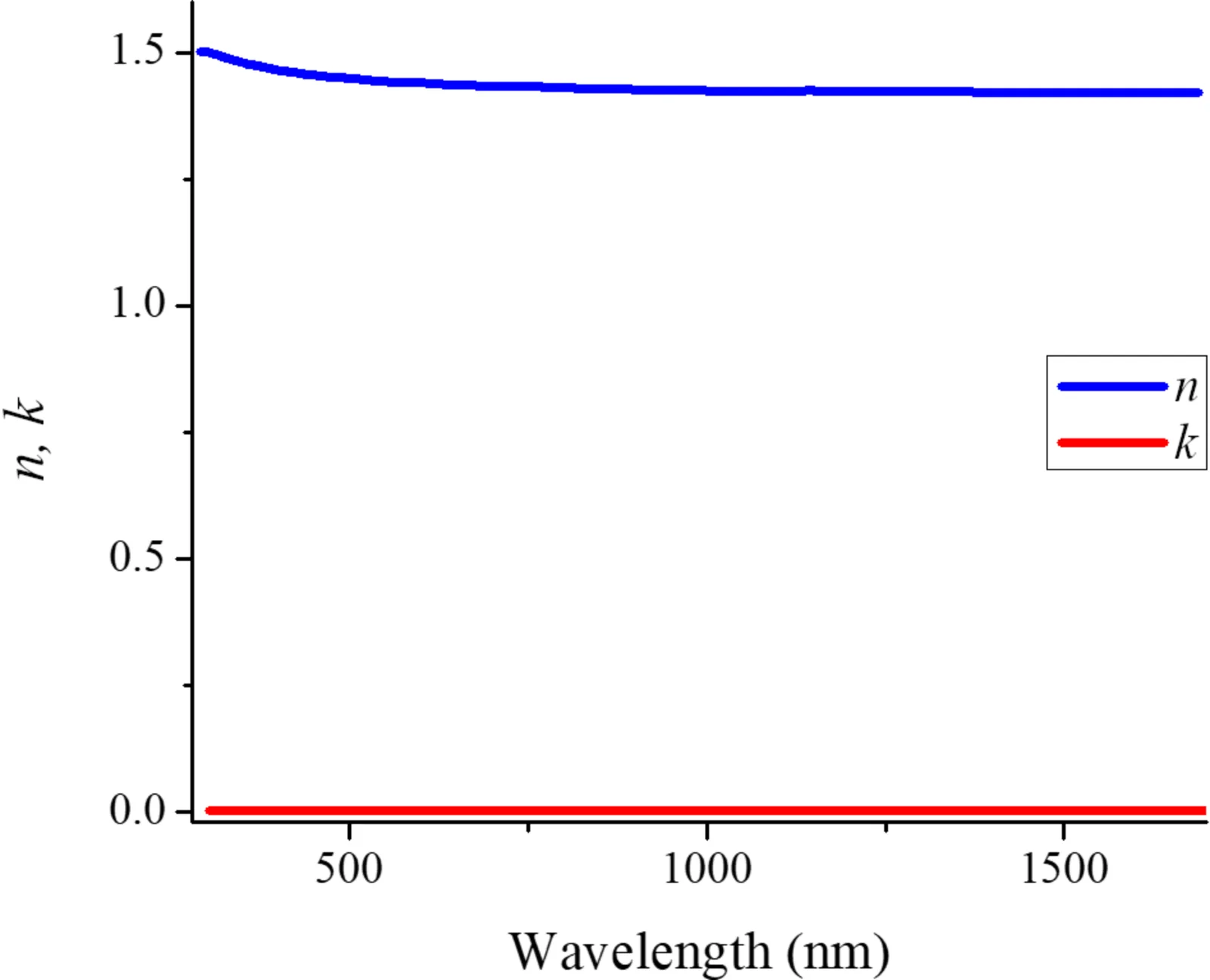
Ellipsometry results for the *n* and *k* values in the range from 300 to 1700 nm of a glass without Au-NPs.

The optical characterization of the glass without Au-NPs was performed in a spectroscopic ellipsometer, covering the wavelength range from UV to NIR (300–1700 nm). The results for refractive index (*n*) and extinction coefficient (*k*) are presented in Figure 9. It shows a refractive index close to 1.45 in the UV–vis–NIR, while *k* remains below the limit of detection of the ellipsometer (0.01). Measurements on glasses containing Au-NPs (not presented here) showed similar *n* and *k* values, because the volume fraction of NPs in the nanocomposite glass is too small to measurably modify its effective optical constants compared with the glass without NPs.

## Conclusion

In this investigation, we demonstrate the synthesis of nanocomposite glasses and gelatins by combining sol–gel and pulsed laser ablation in liquids techniques. Using this combination, we have been able to embed Au nanoparticles in a silicate matrix as well as in commercial gelatin. The properties of the obtained nanocomposites strongly depend on the laser ablation parameters and on the drying procedure. This versatile experimental approach can be implemented using a broad range of targets and precursors to obtain a wide range of nanocomposite materials with tailored optical properties for potential applications in photonics.

## Experimental

**Figure 10:**
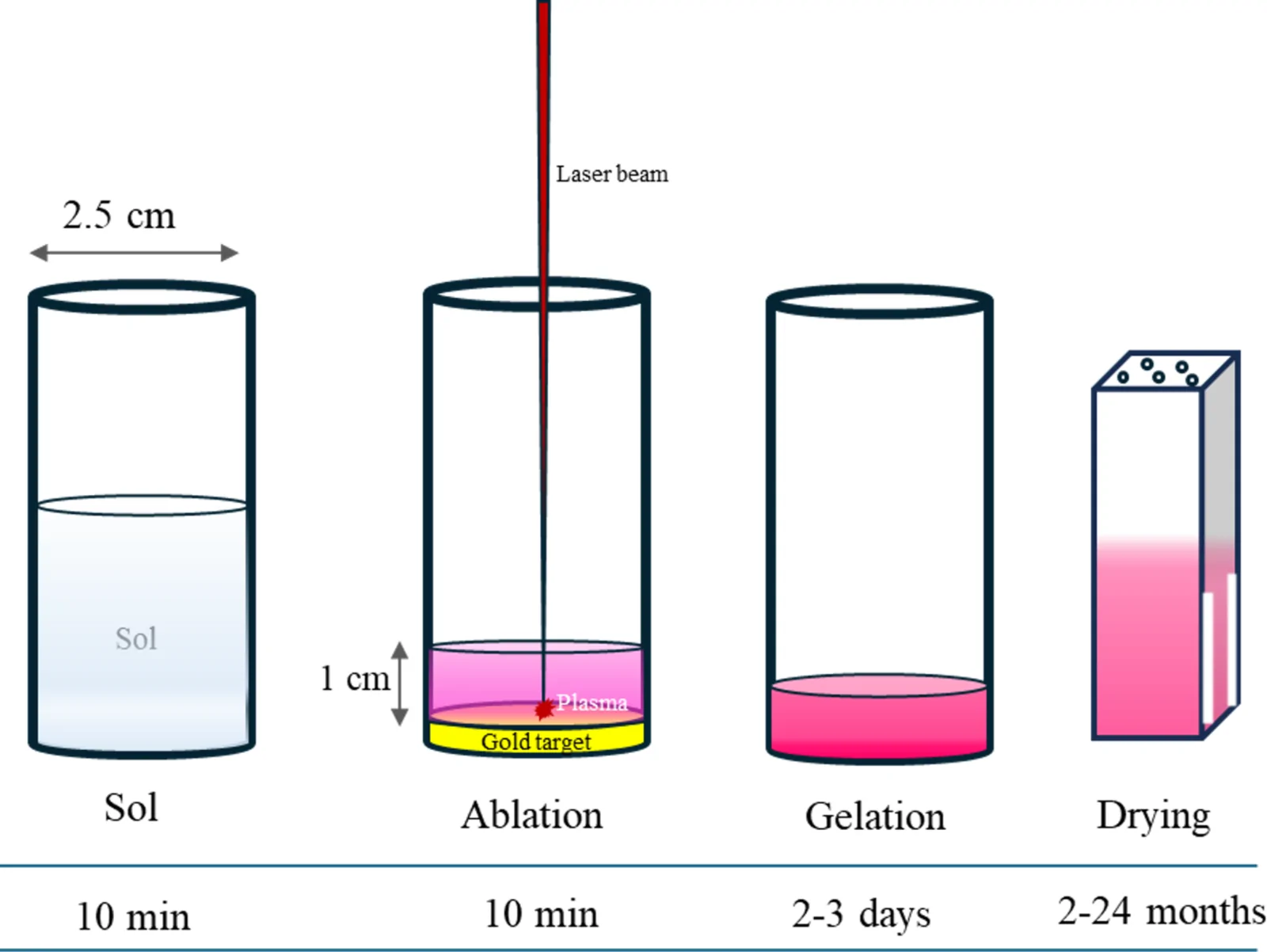
Schematic representation of the synthesis of the nanostructured sol–gel glasses.

The experimental procedure is shown schematically in Figure 10. The reagents used were of analytical grade. The pure (reference) glasses were prepared adding dilute hydrochloric acid as catalyzer to the tetraethyl orthosilicate (TEOS) with a molar ratio of 1:4 (final concentration of 1%). The catalyzer was prepared by adding 0.18 mL of hydrochloric acid to 1.5 mL of deionized water. This was added gradually to 4.74 mL of TEOS under constant stirring until a homogeneous solution was obtained. The sol–gel solution was prepared approximately 1 h before the ablation process. After the ablation, the samples were transferred into 1.5 mL plastic cuvettes for UV spectroscopy and covered partially with parafilm with some holes to let the solvent evaporate.

The solution jellifies after 20 to 30 h and forms a gel that fills the vessel. At this point, the material begins to shrink, which leads to a significant weight loss of up to 30%. This shrinkage occurs over a period of approximately two months. After this process, the sample is a solid block 6 × 2 × 10 mm^3^ in size.

For the case of gelatins, the procedure is almost identical; however, the preparation of the solution is simpler and faster since it involves only gelatin and hot tap water. The ablation process is the same, the gelation time is 1 h, and drying occurs typically in three days.

### Ablation in sol–gel

The nanocomposite glass was prepared laser-ablating a high purity (99.99%) commercial Au target with a diameter of 2.5 cm and thickness of 0.6 cm, submerged in the sol–gel precursor solution. For this purpose, the target was placed inside a glass cylindrical container with an inner diameter slightly larger than the target diameter to fit it at the bottom of the vessel. Afterwards, 5 mL of the sol were added forming a layer with a thickness close to 1 cm over the target surface. The laser beam was directed by means of mirrors and focused onto the target surface through a lens with 12 cm focal length. Laser ablation was carried out using the fundamental emission of a Nd:YAG laser at a wavelength of 1064 nm and 8 ns of pulse duration working at a repetition rate of 10 Hz. Experiments were performed at a laser energy of 100 mJ in a 2 mm diameter laser spot. The ablation time was typically 10 min; this value was determined empirically when observing that the sol turned from transparent to a pink color. The longer the ablation time, the higher the density of nanoparticles; however, the ablation efficiency decreases with time because the suspended Au nanoparticles exert a screening effect on the incident radiation. In the case of gelatins, 1.6 g of commercial pure gelatin were mixed with 62.5 mL of hot (70 °C) tap water under constant stirring for 20 min. Once the temperature decreased to 40 °C, the mixture was poured into the vessel with the Au target for the laser ablation process. The same configuration is used for TEOS and gelatins; however, the preparation parameters are different since the ablation process is more efficient in the gelatin solution than in the TEOS solution. Therefore, for the same energy density, the ablation time was reduced significantly, typically by 50%. In both cases, the presence of gold nanoparticles can be observed readily since the solution exhibits a pink color as shown in Figure 1 and Supporting Information File 1, Figure S1.

The optical properties of the nanocomposites and colloids were characterized by UV–vis spectroscopy with a Varian Cary 2000 double-beam spectrophotometer in transmission configuration at normal incidence. The presence of Au-NPs in the obtained nanocomposites was determined by TEM (JEOL JEM 2100F); for this purpose, the sample was prepared by mechanically grinding in an agate mortar. The powder was placed in a vial with alcohol and ultrasonicated for 30 min to disperse and homogenize it. Afterwards a drop of the obtained suspension was deposited on a formvar-coated Cu grid, allowing the solvent to evaporate completely at room temperature. Specular reflectance was measured with a Woollam VASE ellipsometer. AFM profiles were obtained with a Park XE7 microscope with OML-AC160TS cantilevers in non-contact mode.

## Supporting Information

File 1 Gelatin results and Mie simulations.

File 2 Change of color upon illumination.
Video that illustrates the change of color upon illumination in reflection and transmission in real time for the case of the glass sample that has been drying for two years. Video is also available on: https://youtu.be/WKpvlA15s68.

## Data Availability

Data generated and analyzed during this study is available from the corresponding author upon reasonable request.
