# Supplementary material for: Combining laser ablation and sol–gel techniques for the synthesis of nanostructured organic–inorganic matrices
Source: Beilstein J Nanotechnol. 2026 Oct 23;17:1478–87. doi: 10.3762/bjnano.17.101 (PMC13701152; doi:10.3762/bjnano.17.101)
Supplement: Supplementary file 1 [file Beilstein_J_Nanotechnol-17-1478-s001.pdf]

BEILSTEIN JOURNAL OF NANOTECHNOLOGY

Supporting Information

for

Combining laser ablation and sol–gel techniques for the synthesis of nanostructured organic–inorganic matrices

Emmanuel Haro-Poniatowski, Cesar Augusto Guarín, Luis Guillermo Mendoza-Luna, Luis Escobar-Alarcón, José Luis Hernández-Pozos, L. Irais Vera-Robles, Patricia Castillo, Fátima Cabello, Johann Toudert, Fernando Chacón-Sánchez, Marina García-Pardo, Rosalia Serna, José Gonzalo and Javier Solís

Beilstein J. Nanotechnol. **2026,** *17,* 1478–1487. doi:10.3762/bjnano.17.101

Gelatin results and Mie simulations

License and Terms: This is a supporting information file under the terms of the Creative Commons Attribution License (https://creativecommons.org/licenses/by/4.0). Please note that the reuse, redistribution and reproduction in particular requires that the author(s) and source are credited and that individual graphics may be subject to special legal provisions.
The license is subject to the *Beilstein Journal of Nanotechnology* terms and conditions: (https://www.beilstein-journals.org/bjnano/terms)

S1 Gelatin Results

As in the case of the nanostructured glasses, the PLAL procedure must start before the gelatine has jellified. Just after preparation, the gelatin is liquid and close to 70 °C in temperature. Jellification occurs around 18 °C; therefore, we have incorporated the Au-NPs into the gelatin by PLAL at a temperature around 40 °C and jellification occurred after a few hours.

Drying and shrinking processes in gelatins

Drying and shrinking are different in the case of gelatins compared to the sol–gel glasses. The most interesting results were obtained for gelatins that were dried in Petri dishes. The nanostructured hot liquid was poured into the Petri dish and had a height of 5 mm. After ten days of drying, a flexible thin film 20–50 μm thick was obtained.

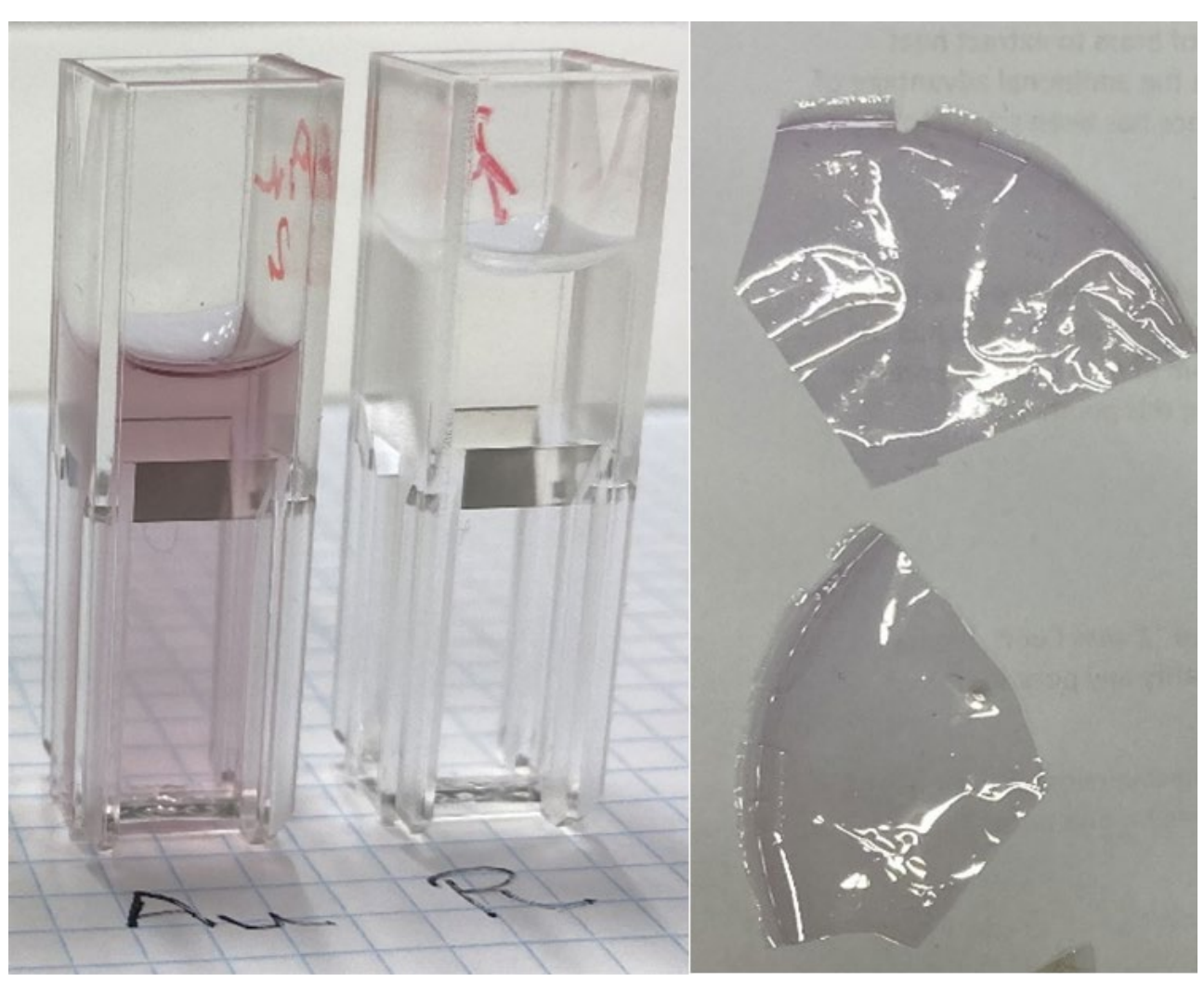

Figure S1: Left: As-prepared gelatin after PLAL (with Au-NPs) and as-prepared gelatin. Right: Dried gelatin films with Au NP-s.

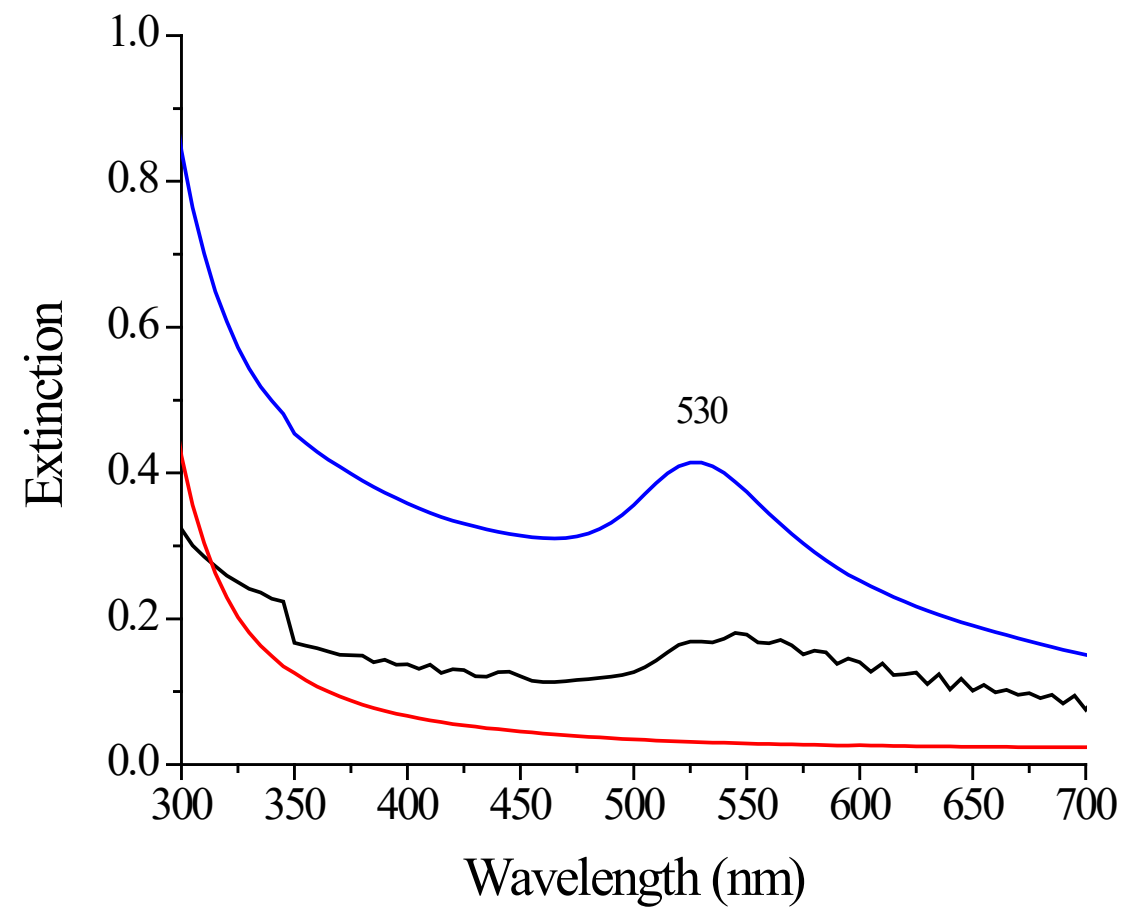

Figure S2: Extinction spectra of gelatins. The blue curve corresponds to the as-prepared gelatin with Au-NPs. The black curve corresponds to the gelatin dried (thin film of gelatin with Au-NPs). Red curve: As-prepared reference gelatin (without Au-NPs).

S2 Mie Simulations

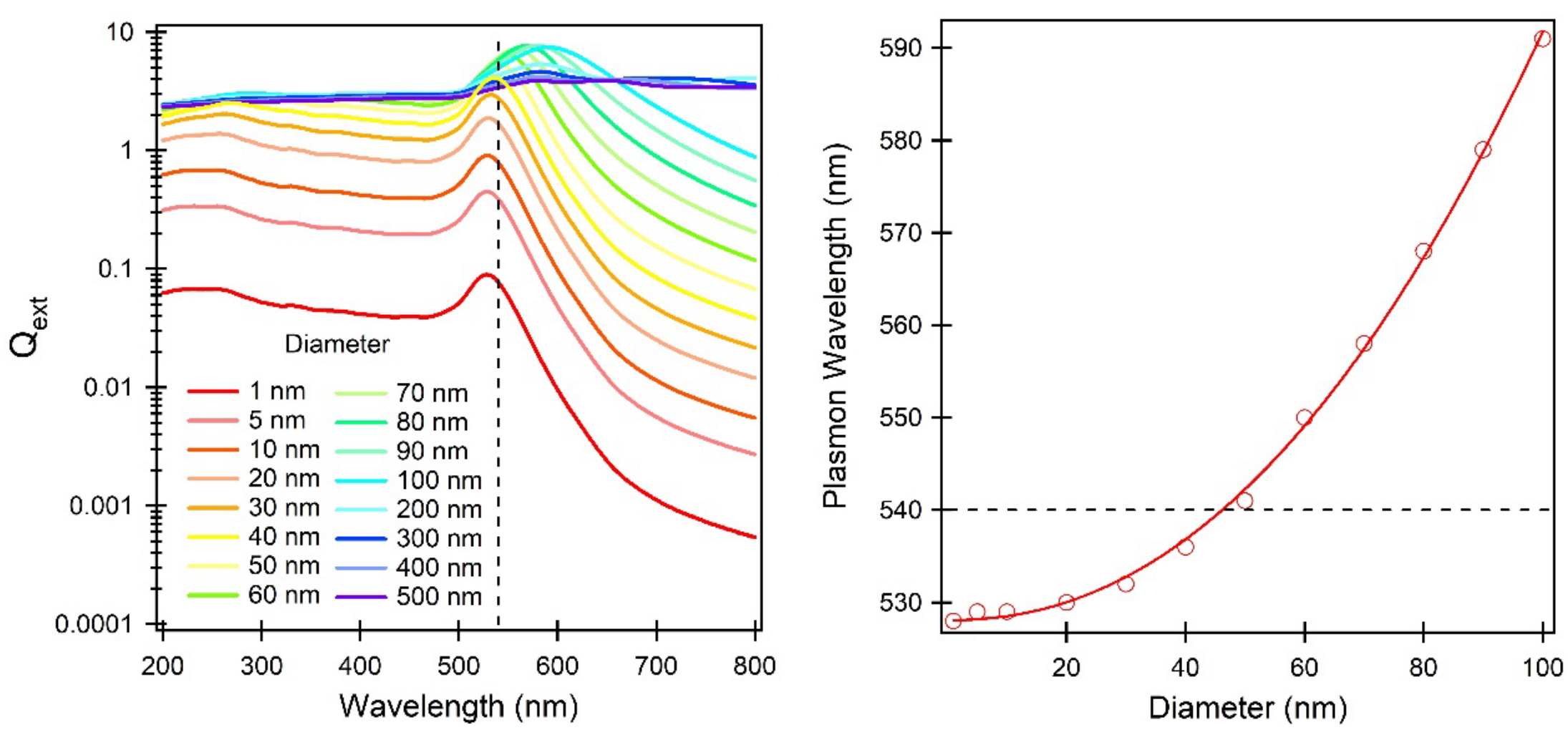

Figure S3: Mie simulations for spherical Au-NPs in a matrix. The NP diameter was varied between 1 and 500 nm. The refractive index of the matrix was 1.44. The obtained extinction spectra (Q_{ext} vs wavelength) are plotted in a log scale on the left graph. The vertical dashed line represents the 540 nm wavelength. The plasmon resonance wavelength is plotted vs the NP diameter on the right graph, where the horizontal dashed line represents the 540 nm wavelength.
